# Systemic effect of sympathectomy in the treatment of localized hyperhidrosis

**DOI:** 10.1007/s13304-025-02163-8

**Published:** 2025-03-10

**Authors:** Mehmet Çetin, Furkan Süral, İlteriş Türk, Necati Solak, Kenan Sönmez, Koray Aydoğdu

**Affiliations:** 1 Department of Thoracic Surgery, Ankara Etlik City Hospital, Ankara, Turkey; 2 Department of Thoracic Surgery, Ankara Atatürk Sanatoryum Training and Research Hospital, Ankara, Turkey; 3 Department of Ophtalmology, Etlik City Hospital, Ankara, Turkey

**Keywords:** Hyperhidrosis, Pupillometry, Sympathectomy

## Abstract

Hyperhidrosis is thought to result from excessive stimulation of sweat glands due to increased sympathetic activity; however, data on systemic responses following bilateral thoracic sympathectomy as the definitive treatment have not yet been sufficiently evaluated. This study, designed as a prospective cohort investigation, included 24 patients who underwent sympathectomy for palmar and axillary hyperhidrosis at our center in 2023, along with an age-matched control group of equal size. In the sympathectomy group, pupillometry measurements were performed 1 day before surgery and on the 7th postoperative day, while the control group underwent measurements at corresponding intervals. Data from the sympathectomy group were analyzed both preoperatively and postoperatively and compared with the control group. According to the Hyperhidrosis Disease Severity Scale, all patients had severe hyperhidrosis. Sympathectomy was performed at the T3 level in 9 patients and at both T3 and T4 levels in 15 patients. The postoperative satisfaction rate was recorded as 95.8%. No statistically significant differences were observed between preoperative pupillometry measurements of the sympathectomy group and those of the control group. However, when comparing preoperative and postoperative data within the sympathectomy group, significant differences favoring increased parasympathetic activity were noted in the latency of contractions and photopic low values (*p* = 0.016 and *p* = 0.038, respectively). Our study is one of the pioneering works to objectively demonstrate, through a quantitative method, that sympathectomy for hyperhidrosis enhances the parasympathetic system response in the ocular region.

## Introduction

Hyperhidrosis is a disorder characterized by excessive sweating, often referred to as a sweating disorder. While hyperhidrosis can affect the entire body, it predominantly involves localized areas such as the hands, feet, and axillae. The primary etiopathogenesis is attributed to excessive stimulation of sweat glands due to heightened sympathetic activity, as no anatomical abnormalities are observed in the sweat glands of these patients [1, 2].

In surgical treatment, the goal is to interrupt the sympathetic chain responsible for the increased local sympathetic activity, thereby halting excessive sweating. It is reported that the success rate of surgery exceeds 95% in hyperhidrosis patients, offering a definitive solution compared to non-surgical treatments. Recent studies have demonstrated high patient satisfaction following endoscopic thoracic sympathectomy (ETS) for primary hyperhidrosis. Gossot et al. found that 85% of patients reported significant improvement in quality of life, with the majority expressing high levels of satisfaction with the procedure's outcome. Their long-term follow-up highlighted the procedure's effectiveness in reducing symptoms of hyperhidrosis, further improving patient well-being [3]. Similarly, Lin and Kuo analyzed the outcomes of ETS in patients with palmar hyperhidrosis and found that 90% of patients reported significant symptom relief and an increase in their overall quality of life. These findings are consistent with those of earlier studies, where patient satisfaction remained high, indicating that ETS continues to be a highly effective treatment for hyperhidrosis [4]. However, post-surgical patients may experience compensatory sweating in areas outside the surgical site, which accounts for a significant proportion of postoperative dissatisfaction [5–7]. In addition, it has been stated that neurological complications such as partial Horner's syndrome may develop following this procedure due to the anatomical proximity of the sympathetic chain to the oculosympathetic pathway [8].

Miosis and mydriasis are physiological pupillary responses associated with the sympathetic and parasympathetic systems. These responses require an intact and functional anatomical structure and neural pathways. Advanced automatic pupillometry devices now enable the acquisition of reproducible and objective data on pupillary functions. Pupillometry facilitates the measurement of pupil diameter and its rate of change based on iris functions under varying light intensities [9].

In our study, we aimed to investigate the relationship between pre- and post-sympathectomy automatic pupillometry measurements, a non-invasive and objective indicator of sympathetic activity, in cases of localized hyperhidrosis.

## Materials and methods

Our study is a prospective cohort study. Prior to the study, local ethics committee approval was obtained (AEŞH-EK1-2023-401) and the research was conducted in accordance with the Declaration of Helsinki. Additionally, informed consent forms were collected from all participants.

The study included patients diagnosed with hyperhidrosis in 2023 who underwent bilateral ETS for primary complaints of palmar and axillary sweating (*n* = 24). These were compared with age-matched individuals without any complaints of local or generalized excessive sweating (*n* = 24). Both groups consisted of patients without comorbid conditions. Patients lost to follow-up or those whose pupillometry measurements were unavailable were excluded from the study.

Endoscopic sympathectomy was performed under general anesthesia and bilaterally in all patients. Following anesthesia induction, a double-lumen endotracheal tube was placed. After occluding the right lung, the thorax was explored through a 1 cm incision made in the right anterior axillary area, using a 30-degree 2.9 mm optical scope, and the paravertebral sympathetic chain was visualized with the assistance of a hook. Subsequently, the T3 or T3–T4 sympathetic chains were cut according to the surgeon's preference. In our clinic, surgeons routinely perform T3 or T3–4 sympathectomy. In this study, since the procedure was performed at the T3 level in every patient, no restrictions were applied regarding this. Additionally, alternative pathways were cauterized over the rib upper Knutz fibers. A catheter was then inserted through the thoracic incision, connected to a closed water-sealed system, and blockage was requested to be terminated. After ensuring full lung expansion, the catheter was removed. The incisions were then closed primarily. The same procedure was subsequently performed for the left lung. Postoperative chest radiographs were performed on all patients. Surgical intervention was applied to symptomatic patients with detected pneumothorax. Additionally, if the chest radiograph taken the following day was normal, discharge was planned for the patients.

Initially, demographic characteristics, medical histories, sweating areas, medication use, sympathectomy level performed, surgical duration, complications, chest tube application, length of hospital stay, reflex sweating, satisfaction post-surgery, and follow-up durations were evaluated for all patients. All patients underwent single-port bilateral ETS via a 2 cm incision in the axillary region. No chest tubes were inserted during the surgery. Postoperative symptomatic pneumothorax cases requiring chest tube placement were considered complications. Surgical satisfaction was assessed by asking, "Would you choose to undergo the surgery again if in the same situation?" A "yes" response was considered indicative of satisfaction.

The intensity of sweating before and after surgery was evaluated using the Hyperhidrosis Disease Severity Scale (HDSS):

- Group 1: "My sweating is never noticeable and never interferes with daily activities."
- Group 2: "My sweating is tolerable but sometimes interferes with daily activities."
- Group 3: "My sweating is barely tolerable and frequently interferes with daily activities."
- Group 4: "My sweating is intolerable and always interferes with daily activities."

Groups 1 and 2 were classified as mild to moderate hyperhidrosis, while groups 3 and 4 were classified as severe hyperhidrosis. A reduction of at least two groups was considered a significant response to treatment and also postoperative patient satisfaction. Compensatory sweating was also evaluated using the same scale, with groups 3 and 4 considered significant compensatory sweating [10].

All patients and control group participants underwent routine ophthalmic examinations. All measurements were conducted by the same experienced technician using an automated quantitative pupillometry device (MonPack One, Vision Monitor System, Metrovision, Perenchies, France). This system features a near-infrared illumination and a high-resolution camera (880 nm) capable of simultaneous measurement of both eyes in complete darkness. Pupillometry measurements were performed using white light stimuli generated by combining blue (465 nm), green (523 nm), and red (632 nm) LEDs. The device enabled precise static and dynamic pupillometry measurements with an accuracy of 0.1 mm. Measurements were performed sequentially for all participants, and average values were used for analysis. To minimize observer-related errors, measurements were performed in the device's automatic detection mode, and only high-quality measurements were recorded. To account for diurnal variations in pupillary response, all measurements were conducted under identical environmental conditions between 12:00 and 15:00. Participants were instructed to maintain stable fixation on a target during testing. The data obtained under these conditions were used for statistical analysis.

Static pupillometry tests were conducted under varying illumination conditions: scotopic (0.1 cd/m^2^), mesopic (1 cd/m^2^), low photopic (10 cd/m^2^), and high photopic (100 cd/m^2^) settings. Following a 5-min dark adaptation period, dynamic pupillometry measurements were taken in darkness using white light flash stimuli for 90 s. These stimuli were presented at 200 ms ON and 3300 ms OFF intervals, with a total brightness of 100 cd/m^2^. The average response to visual stimuli was measured using the following parameters: resting pupil diameter, pupil contraction latency, amplitude, duration, speed, dilation latency, dilation duration, and dilation speed. Only high-quality measurements from images taken at a 5° angle to the fixation axis were analyzed. The automated analysis software of the device was used to record and analyze static and dynamic pupillometry measurements.

Individuals with a history of thoracic surgery or complaints of localized or generalized excessive sweating were excluded. Statistical comparisons were performed between the surgical and control groups, as well as between preoperative and postoperative measurements within the surgical group.

### Statistical analysis

All analyses were conducted using SPSS 24.0 software. Descriptive statistics included frequency (n), percentage (%), and median (min–max) values for age, hospital stay, and follow-up duration. Due to the sample size being under 30, nonparametric tests were used. Between-group comparisons were performed using the Mann–Whitney U test, while within-group comparisons were conducted using the Wilcoxon signed-ranks test. A p value of less than 0.05 was considered statistically significant.

## Results

The study included 24 patients in the hyperhidrosis group who underwent ETS and 24 participants in the control group. The variable data for all participants are presented in Table 1. Among the patients who underwent sympathectomy, all reported preoperative palmar hyperhidrosis and seven patients (29.2%) additionally reported axillary sweating.

**Table 1:** Comparison of variables between the patient and control groups

	Patients (hyperhidrosis)		Control group	
	Number (n, %)	Median (min–max)	Number (n, %)	Median (min–max)
Age		21 (16–43)		25 (23–26)
Gender				
Male	10 (%41,7)		10 (%41,7)	
Female	14 (%58,3)		14 (%58,3)	
Drug use				
Present	4 (%16,7)		0 (%0)	
Absent	20 (%83,3		24 (%100)	
Surgical history				
Present	5 (%20,8)		0 (%0)	
Absent	19 (%79,2)		24 (%100)	
Chronic disease				
Present	5 (%20,8)		0 (%0)	
Absent	19 (%79,2)		24 (%100)	
Surgical duration (minute)		42 (35–62)		
Length of stay (day)		2 (1–2)		
Follow-up (month)		12 (2–17)		

Preoperative HDSS scores for the sympathectomy group showed that 17 patients (70.8%) had a score of 4, while 7 patients (29.2%) had a score of 3, indicating that all patients had severe hyperhidrosis. Regarding the levels of sympathectomy performed, 15 patients (62.5%) underwent sympathectomy at levels 3 and 4, while 9 patients (37.5%) underwent sympathectomy only at level 3.

Postoperatively, reflex sweating was observed in 18 patients (75%). The areas affected by reflex sweating included the back in 11 patients (45.9%), the abdomen and legs in 4 patients each (16.7%), and the feet in 3 patients (12.5%). Compensatory sweating was evaluated postoperatively, with HDSS scores of 1 observed in 23 patients (95.8%) and a score of 3 in 1 patient (4.2%).

While no complications were observed intraoperatively, two patients (8.3%) experienced pneumothorax as a complication following sympathectomy. One patient required 28F chest drainage, and the other underwent closed underwater drainage with a 12F pleural catheter. As the lungs were fully expanded after the procedure and no findings suggestive of pneumothorax were observed on the follow-up chest X-ray 1 day later, the drainage and catheterization were terminated. Patient satisfaction following sympathectomy was reported at 95.8%.

**Fig. 1:**
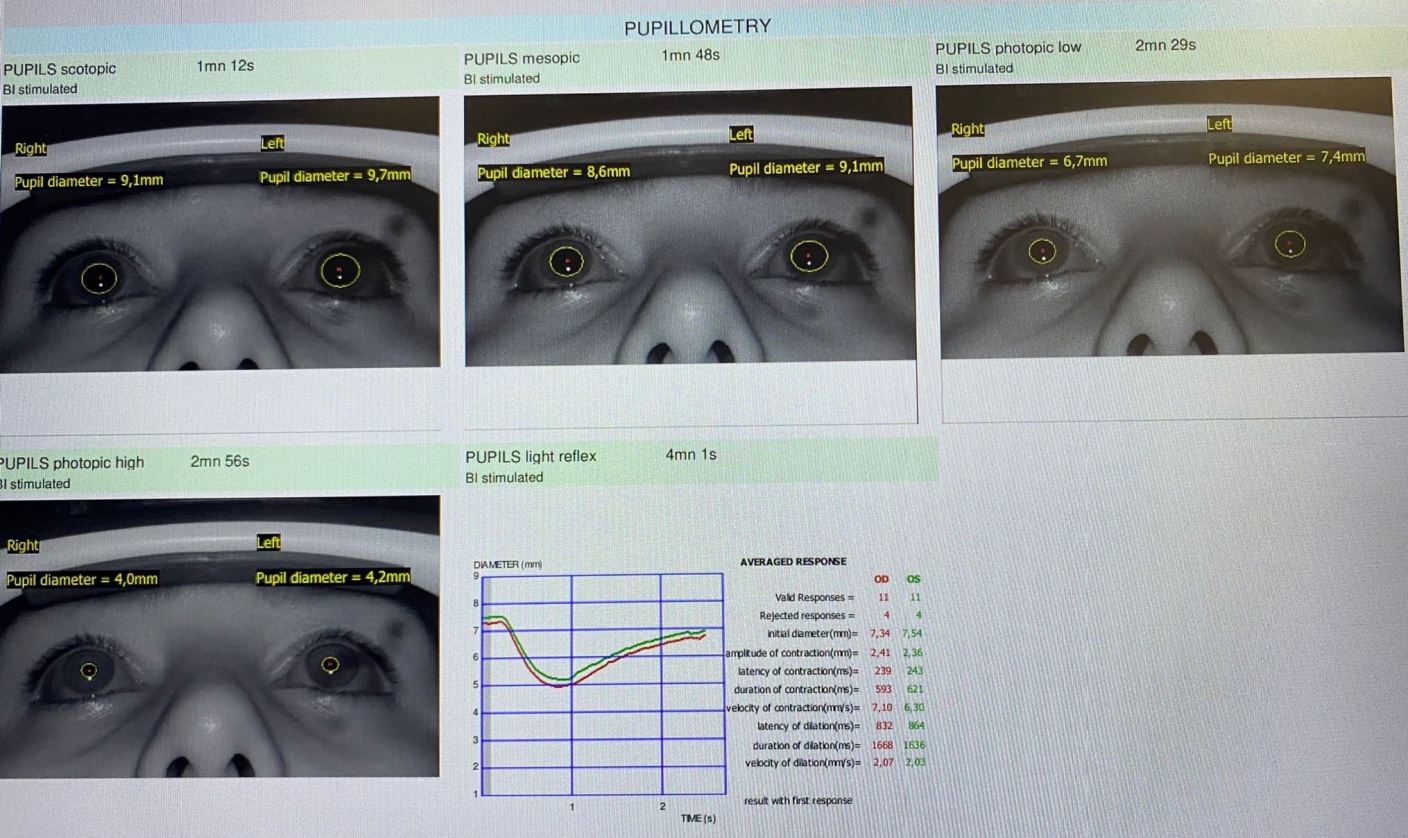
Preoperative automatic pupillometry image

**Fig. 2:**
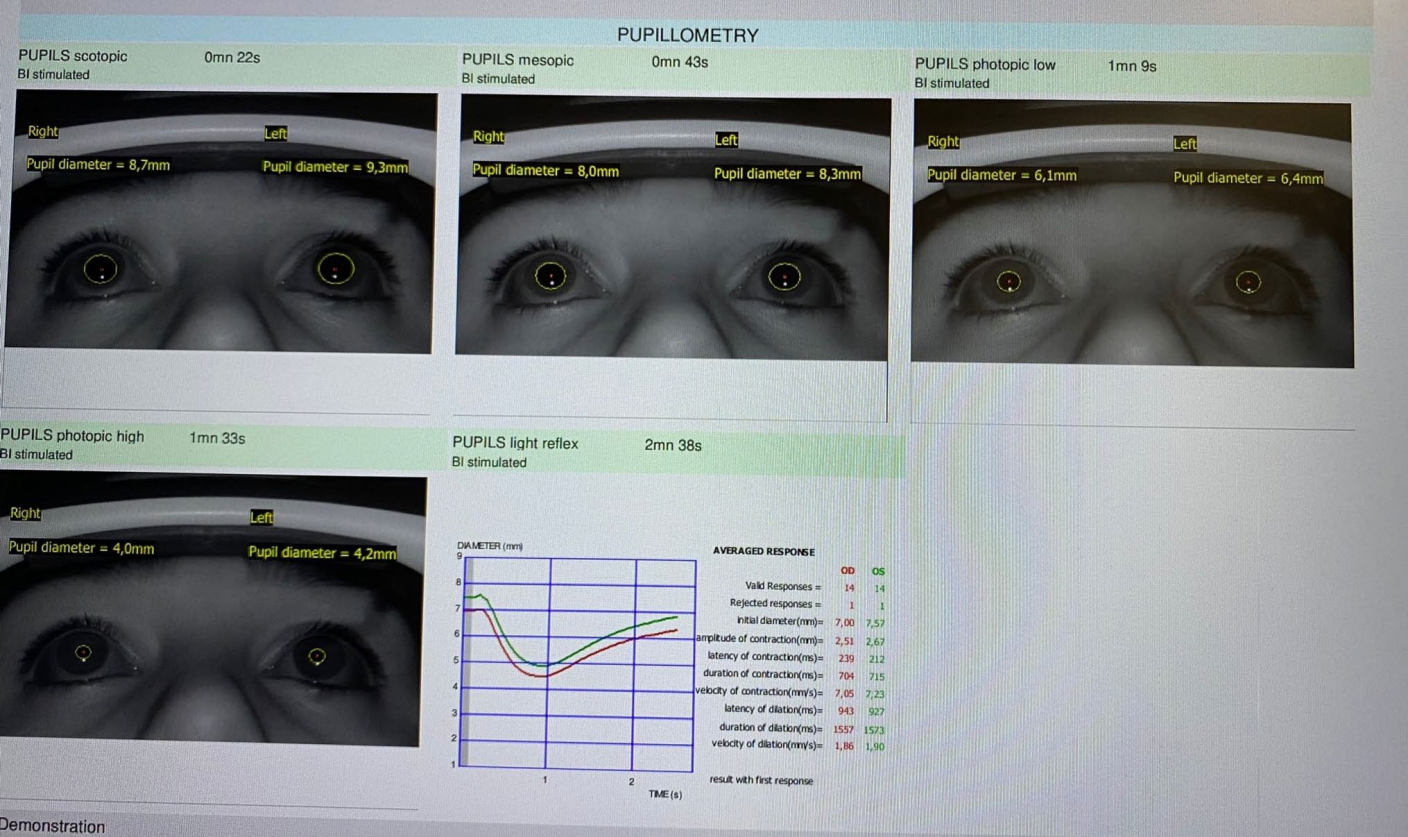
Postoperative automatic pupillometry image

Comparisons of preoperative and postoperative pupillometry measurements in hyperhidrosis patients, as well as the measurements of the control group, are presented in Table 2. Also preoperative and postoperative automatic pupillometry measurements of a patient are shown in Figs. 1 and 2.

**Table 2:** Comparison of patients’ preoperative measurements with their postoperative measurements within the same group and separately with the control group

	Hyperhidrosis preoperative (mean, SD)	Hyperhidrosis postoperative (mean, SD)	*p*	Hyperhidrosis preoperative (mean, SD)	Control group (mean, SD)	*p*
Valid responses	10,63 (3,62)	11,08 (3,43)	0,587	10,63 (3,62)	9,25 (3,76)	0,188
Rejected responses	4,75 (3,58)	4.,42 (3,32)	0,613	4,75 (3,58)	5,92 (3,79)	0,291
Initial diameter (mm)	6,23 (0,71)	5,98 (0,63)	0,140	6,23 (0,71)	6,14 (0,56)	0,942
Amplitude of contractions (mm)	1,92 (0,29)	1,89 (0,29)	0,131	1,92 (0,29)	2,06 (0,34)	0,543
Latency of contractions (ms)	238,54 (34,20)	222,46 (42,07)	**0,016**	238,54 (34,20)	235,54 (33)	0,628
Duration of contractions (ms)	595,58 (66,61)	598,25 (61,95)	0,853	595,58 (66,61)	593,75 (60,28)	1,000
Velocity of contractions (mm/s)	6,09 (0,89)	6,05 (0,95)	0,668	6,09 (0,89)	6,53 (1,28)	0,409
Latency of dilatations (ms)	834,13 (46,32)	820,83 (43,01)	0,281	834,13 (46,32)	829,29 (56,27)	0,606
Duration of dilatations (ms)	1638,67 (74,41)	1657,38 (47,01)	0,277	1638,67 (74,41)	1631,88 (94,85)	0,781
Velocity of dilatations (mm/s)	1,87 (0,32)	1,81 (0,30)	0,230	1,87 (0,32)	1,88 (0,30)	0,984
P. scotopic	7,48 (0,86)	7,55 (0,79)	0,405	7,48 (0,86)	7,52 (0,56)	0,812
P. mesopic	6,21 (1,44)	5,93 (1,24)	0,053	6,21 (1,44)	5,89 (1,05)	0,348
P. photopic low	4,70 (1,16)	4,40 (0,87)	**0,038**	4,70 (1,16)	4,34 (0,59)	0,457
P. photopic high	3,25 (0,44)	3,15 (0,37)	0,112	3,25 (0,44)	3,14 (0,34)	0,325

## Discussion

Evaluation of pupil diameter is described as a practical, useful, and non-invasive method for analyzing the autonomic nervous system [11]. For this purpose, objective measurements and evaluations of pupil reflexes can be made using automatic pupillometry, allowing analysis of sympathetic and parasympathetic system innervation through defined parameters [12]. Various analyses have been conducted across different disease groups, including bipolar disorder, Alzheimer’s disease, and age-related autonomic nervous system responses in healthy individuals, and the results have been used as objective criteria to assess the balance between the sympathetic and parasympathetic systems [9, 12–15]. In their study, Ramos et al. evaluated pupillary responses following T3 sympathectomy surgery and demonstrated the development of subclinical Horner's syndrome, which they associated with the anatomical proximity of the sympathetic chain to the oculosympathetic pathway [8]. In our study, there was no significant difference between the preoperative pupillometry measurements of the hyperhidrosis group and the control group. However, significant differences were observed in the latency of contractions and photopic low values between the preoperative and postoperative pupillometry measurements in the patient group, indicating increased parasympathetic system activity. This study, being one of the first on the subject, is significant in showing that the systemic effects of the ETS, a treatment for hyperhidrosis, can arise even though hyperhidrosis is a disease directly related to sweat glands. We believe that the sympathetic activity in the face might have decreased due to the level of sympathectomy. Therefore, we believe that comparing pupillometric responses in isolated T3 and isolated T4 sympathectomies in the following stages may provide additional insights into the topic.

Hyperhidrosis, although a benign disease, significantly affects the quality of life and therefore requires treatment. Although medical treatments, such as botulinum toxin, exist, the only permanent and definitive solution for patients without underlying diseases or conditions contraindicating surgery is thoracic sympathectomy [16, 17]. Additionally, surgical treatment is preferred due to its low morbidity and mortality, rapid recovery process, and immediate postoperative response [1]. In our clinical approach, we prioritize the social anxiety and professional life impact of hyperhidrosis when making treatment decisions. We also ensure the identification of the underlying pathophysiologies that might contribute to sweating and consult endocrinology and cardiology for the preoperative evaluation of rare postoperative complications such as arrhythmias. Postoperative hospital stay was 2 days, and only two patients required postoperative chest tubes and pleurovac placement, which aligns with the low morbidity seen in hyperhidrosis cases.

In patients with hyperhidrosis, the surgical success rate is evaluated based on symptom control and patient satisfaction. A review published in 2021 reported a 96% rate of symptom control and a 92% overall satisfaction rate for primary hyperhidrosis cases. It was noted that patient satisfaction decreased to 93.22% in the first postoperative evaluation, and by 12 months, it decreased further to 88.23% [18]. In Toolabi’s study, evaluating 10-year results, near-complete positive outcomes were observed in palmar hyperhidrosis cases, while satisfactory results were noted for craniofacial hyperhidrosis. However, in cases of pure axillary hyperhidrosis, results were poor enough to suggest discontinuing the surgical procedure [19]. In our study, when asked, “If we were to start the operation again, would you still choose to undergo it?”, all patients answered “yes.” The patient satisfaction rate was 95.8%. Only one patient expressed dissatisfaction, with the primary issue being compensatory sweating. In this patient, Grade 3 and widespread compensatory sweating occurred. However, symptom control was 100% in this patient. We believe that our strict patient selection criteria played an important role in achieving these results. In our clinical approach, all patients were classified as Grade 3 or 4, with the exception of seven patients with palmar–axillary hyperhidrosis involving the palms. Therefore, it can be stated that late-stage symptom control was maintained at the same rate due to the average follow-up period of 12 months.

In satisfaction assessments, although the surgical morbidity and mortality for hyperhidrosis are low, the primary cause of postoperative dissatisfaction in patients is compensatory sweating [20]. Compensatory sweating (CS) is the most common complication, occurring in 3–98% of cases. Predictors for CS include older age, higher body mass index, elevated body temperature, family history, and smoking, according to various publications [12, 21, 22]. While some studies suggest that patients under 25 are ideal surgical candidates, others report better outcomes in older patients, and yet others find no significant relationship [19, 21, 23, 24]. There are several hypotheses regarding the mechanism of compensatory sweating. It is suggested that when the sympathetic chain is cut, sweating attempts to compensate in other areas of the body, meaning the total amount of sweating does not change. Additionally, it is thought that the block of negative feedback signals following the severing of the sympathetic ganglion leads to increased sweating in other regions through augmented efferent signals [25, 26]. Despite varying group outcomes in our study, compensatory sweating was observed in 75% of cases, although it did not significantly affect patient satisfaction. The majority of patients with CS were in Group 1, and this did not substantially alter their satisfaction levels, which makes the results encouraging.

For effective hyperhidrosis treatment, it is necessary to target levels below T2, ideally including T3–4 levels, as the Kuntz fibers form at these levels and extend to the brachial plexus [27]. However, research has primarily focused on how patient risk factors, along with surgical factors, influence CS outcomes. A review published in 2008 suggested no relationship between sympathectomy level and CS, but all other studies found that blockage at levels lower than T2 leads to better CS outcomes and a significant improvement in quality of life [1, 25, 27–31]. In our study, all patients received T3 and commonly T3–4 sympathectomy, in line with the literature. Only one patient experienced Grade 3 widespread compensatory sweating, which can be seen as a positive reflection of performing sympathectomy further from T2.

The main value of our study lies in it being one of the first to evaluate the sympathetic system activation through non-invasive and objective pupillometry measurements, in relation to the effectiveness of hyperhidrosis surgery and CS levels. Additionally, the lack of differences in preoperative measurements between the hyperhidrosis and control groups supports the idea that specific sympathetic activity increases in sweat glands in the pathophysiology of sweating.

A major limitations of our study are the sensitivity of dynamic pupillometry measurements and the absence of long-term pupillometry results. Moreover, as only a T3-level sympathectomy was performed on all patients, it hindered the evaluation of the relationship between the level of sympathectomy and pupil response.

In conclusion, hyperhidrosis is a pathology that directly affects the sweat glands and is related to sympathetic activity. The findings from postoperative pupillometry, indicating increased sympathetic activity, demonstrate that although hyperhidrosis specifically affects the sweat glands, the surgery has a systemic effect on the body's overall sympathetic response. The results of this study, along with broader patient group data and further studies on pathogenesis and compensatory mechanisms, may yield more meaningful conclusions.

## Data Availability

The datasets used and/or analyzed during the current study are available from the corresponding author on reasonable request.
